# Advanced Endoscopy Training in the United States: An Advanced Fellow's Perspective

**DOI:** 10.14309/crj.0000000000000612

**Published:** 2021-06-02

**Authors:** Ramzi Mulki

**Affiliations:** 1 Division of Gastroenterology and Hepatology, Department of Medicine, The University of Alabama at Birmingham, AL

## INTRODUCTION

Advanced endoscopy (AE), also known as therapeutic or interventional endoscopy, is an exciting, technologically advanced, and rapidly evolving field of gastroenterology (GI). This editorial aims to offer insight into the process of applying and training in AE in the United States for aspiring and current AE trainees (AET) from the perspective of an AE fellow.

## THE MATCH

Acquiring the skills needed to safely perform AE procedures requires additional training after GI fellowship. The current structure of most GI fellowships lacks adequate hands-on exposure to endoscopic retrograde cholangioscopy (ERCP) and endoscopic ultrasound (EUS) to allow fellows to pursue an academic career dedicated to AE. Therefore, in 2012, the American Society of Gastrointestinal Endoscopy (ASGE) established a standardized application and match process for the AE fellowship (AEF). Most AEF programs participate in “the Match” with some offering positions outside it. The duration of training is 12 months, with some programs extending beyond that for additional training in other procedures and/or research.^1–3^ AE remains a competitive field of GI; between 2012 and 2020, the average applicant match rate was 57%.^4^

## THE RIGHT FIT

The ASGE Match website contains information regarding each program's procedural volume and types, duration of training, faculty, research, and other application/service requirements. There has been a steady increase in the number of programs participating in the Match. In 2020, 63 programs participated in the Match offering a total of 71 positions.^4^ So, how can one find the best fit?

Procedural numbers were previously viewed as surrogates for competence; however, this has changed. Instead of absolute numbers, competence-based thresholds should be achieved before overall proficiency is assessed. The ASGE recommends a minimum of 200 and 225 supervised independent ERCPs and EUS, respectively, before assessing competency.^5^ A multicenter prospective study showed that the average procedural volume to achieve competency in ERCP and EUS was 225 and 250 cases, respectively, using the evidenced-based EUS and ERCP Skills Assessment Tool.^6,7^ Surveys administered to AET in 2019 and 2020 revealed that 75% and 64.1% of trainees achieved > 300 ERCPs and EUS procedures, respectively, during their AEF training year.^4^ Most programs are, therefore, able to offer adequate procedural numbers, and instead, one should focus on other critical aspects of training such as mentorship, diversity and complexity of cases, and placement after fellowship.

Acquiring the cognitive skills of AE is as crucial as learning the technical skills. The ASGE training committee has published a series of core curriculum documents in various procedures such as ERCP, EUS, peroral endoscopic myotomy, endoscopic mucosal resection, endoscopic submucosal dissection, luminal stenting, and ablative therapies to assist AEF programs.^8–14^ Therefore, gauging programs' philosophy on training should be an essential part of evaluating any AEF.

The program should allow time and opportunities for inquiry, endoscopy-focused research, and quality improvement. Identifying a potential mentor is a fundamental aspect that must not be overlooked. The mentee-mentor relationship will continue long after fellowship. Ge et al demonstrated the critical role the mentors played during the endoscopist first independent endoscopic submucosal dissection as a junior faculty out of AEF, with frequent debriefings before, during, and after the procedure, allowing the endoscopist to make significant improvements in technique and strategy.^15^

Applicants must realize that within ERCP and EUS, there is a wide array of therapeutic options. Historically, AET are exposed to biliary ERCP, with less exposure to pancreatic ERCP, advanced cannulation techniques (eg, needle-knife), and interventional EUS.^2^ Opportunities in these areas should be sought after, once basic ERCP and EUS skills have been achieved. ERCP is stratified into 3 grades based on difficulty, with grade 3 being the most difficult (eg, cholangioscopy, pancreatoscopy, and intrahepatic bile duct stone management).^8^ Moreover, there is an expanded array of therapeutic endoscopic procedures, beyond ERCP and EUS. These include areas such as EBT, third space endoscopy, endoscopic mucosal resection, ablative therapies, device-assisted enteroscopies, endoluminal stenting, advanced closure techniques (eg, suturing), and the emerging endo-hepatology.^16^ Training in these areas can be achieved with hands-on experience within the program, simulators and animal models, or seeking elective opportunities at other programs. Therefore, it is essential to be able to identify an area of interest and understand how programs will help achieve training in said interest.

Many AEF programs require service time from the AET to support their salary, while others are able to fund the position. While evaluating programs, the amount of time and type of service required should be taken into consideration. Requirements may include covering general GI consult service, call, outpatient procedures, and/or clinics. It should be noted that although these requirements may not be appealing to many, one should look at the positive aspects. These include maintaining adequate procedural volumes, performing bread and butter endoscopic procedures such as screening colonoscopy, establishing oneself as a new junior consultant, networking with other specialists, and becoming involved in fellow education and mentorship.

## HOW TO PREPARE FOR AEF?

A steep learning curve is seen when transitioning from GI fellowship to AEF. The AET's role lies at the intersection of a general GI consultant, an educator/mentor for GI fellows, a trainee, and liaison for interdepartmental communications.

Preparing for AEF will undoubtfully give a leg up to the busy year ahead. Time management during the final year of GI fellowship is essential to understanding and sharpening cognitive aspects of pancreaticobiliary disorders, as well as becoming familiar with the National Comprehensive Cancer Network guidelines on GI malignancies, and other societal guidelines. Any exposure to EUS and ERCP will help accelerate and fine tune skills throughout this year. Many endoscopy-based societal resources, reviews, and books, as well as videos, have been published and, if started early on, can assist in jump starting the year.

AET should consider communicating in advance with their future program directors and mentors. This will allow an open discussion about training goals, expectations, and an opportunity to discuss potential research projects.

## NAVIGATING THE AEF YEAR

**Figure 1.**
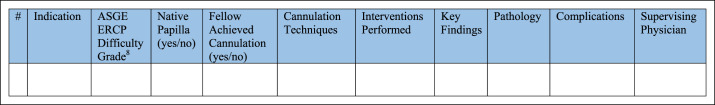
Endoscopic retrograde cholangioscopy procedure log.

**Figure 2.**
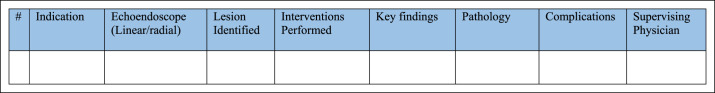
Endoscopic ultrasound procedure log.

The AEF year is a quick one; from learning the logistics, procedures, starting on research, finding a job, and preparing for the GI boards, 6 months would have already passed. Therefore, time management is key to success. Programs have different methods in tracking and assessing competence; nonetheless, AET should get into the habit of tracking their own performance by keeping a procedure log. The log must contain useful information that the AET can refer back to at any point. For example, the log should contain information such as achieving native cannulation, completing sphincterotomy, stent deployment, adequacy of tissue acquisition, and complications, as suggested in Figures 1 and 2. The log can also serve as a reference for potential teaching cases, publications, or discussions during morbidity/mortality conferences. The suggested procedure log should complement any institutional-specific or evidence-based competence assessment tools.^6^

AET should familiarize themselves with novel endoscopic equipment, learn how to use and trouble shoot them should problems occur. It goes without saying that preparing for cases by reading radiological and fluoroscopic images will enable the AET to approach cases thoughtfully. Moreover, AE lies in a unique position between medicine, surgery, pathology, and radiology. Therefore, AET should seek opportunities to participate in multidisciplinary meetings such as tumor board. These meetings have an educational benefit, allow for research collaboration, and, more importantly, improve patient outcomes. AET should actively participate in AE rounds. Moreover, as the year progresses, trainees should seek opportunities to lead rounds, as junior AE faculty, and assist in decision-making and managing complications. As with all other disciplines in medicine, it is important to maintain a good work-life balance and seek opportunities to avoid burnout.

## OPPORTUNITIES OUTSIDE THE ENDOSCOPY SUITE

The novel coronavirus disease of 2019 pandemic has fundamentally impacted the training of procedure-based specialties such as GI. This allowed AEF directors and GI societies to think of innovative methods for endoscopic teaching, many of which will likely continue even as restrictions ease.^17^ Opportunities include hands on models and simulators, endoscopic video rounds, and attending virtual conferences, which are often free of charge to trainees. Finally, connecting and collaboration has been enhanced by many social media platforms such as Twitter.

## FUTURE DIRECTIONS IN ADVANCED ENDOSCOPY TRAINING

The landscape for AET continues to evolve. With more therapeutic endoscopic procedures becoming available, the need for training has increased. Assessing the need for such subspecialties, focusing AEF into specific areas (eg, third space endoscopy), nontraditional routes of endoscopic fellowship training, standardizing AE training across sites, and incorporating AE training to the final year of GI fellowship are some areas that require further investigation.

A final message to AET and prospective applicants: congratulations on reaching this point in your career. AE is an exciting field that will enable you to become pioneers in the future. Finally, this year is an investment on your behalf, for your training; take full advantage of it, and enjoy it to the best of your ability. You will be glad you did!

## DISCLOSURES

Author contributions: R. Mulki wrote and approved the manuscript. R. Mulki is the article guarantor.

Financial disclosures: None to report.
